# Job strain and sense of coherence: Associations with stress-related outcomes among teachers

**DOI:** 10.1177/14034948211011812

**Published:** 2021-05-12

**Authors:** Joacim Ramberg, Sara Brolin Låftman, Jannike Nilbrink, Gabriella Olsson, Susanna Toivanen

**Affiliations:** 1 Department of Special Education, Stockholm University, Sweden; 2 Department of Public Health Sciences, Centre for Health Equity Studies (CHESS), Stockholm University/Karolinska Institutet, Sweden; 3 School of Health, Care and Social Welfare, Mälardalen University, Sweden

**Keywords:** Teacher stress, depressed mood, job strain, sense of coherence, psychological demands, job control, contextual, multilevel

## Abstract

*Background:* Teachers constitute an occupational group experiencing high levels of stress and with high sick-leave rates. Therefore, examining potentially protective factors is important. While prior research has mainly focused on the link between teachers’ own experiences of their work environment and stress-related outcomes, it is also possible that colleagues’ perception of the work environment and their possibilities for dealing with work-related stress contribute to influencing individual teachers’ stress. *Aim:* The aim of this study was to investigate how teachers’ reports of high job strain (i.e. high demands and low control) and sense of coherence (SOC), as well as the concentration of colleagues reporting high strain and high SOC, were associated with perceived stress and depressed mood. *Methods:* The data were derived from the Stockholm Teacher Survey, with information from two cross-sectional web surveys performed in 2014 and in 2016 (*N*=2732 teachers in 205 school units). Two-level random intercept linear regression models were performed. *Results:* High job strain at the individual level was associated with higher levels of perceived stress and depressed mood, but less so for individuals with high SOC. Furthermore, a greater proportion of colleagues reporting high SOC was associated with lower levels of perceived stress and depressed mood at the individual level. ***Conclusions:* High SOC may be protective against work-related stress among teachers. Additionally, the proportion of colleagues reporting high SOC was related to less individual stress, suggesting a protective effect of school-level collective SOC.**

## Introduction

Teachers are an occupational group that experiences high levels of stress in their workplace compared to many other occupational groups [1,2]. This is particularly evident in Sweden where teachers report very high stress levels [3–5], possibly contributing to the high sick-leave rates among teachers in Sweden. Results from several reports also show that the levels of stress among Swedish teachers have increased sharply over the last decades [3,4]. This demanding work situation for teachers at the same time as there is an acute national shortage of teachers makes it important, on a societal level, to increase the knowledge about what factors may contribute to stress and what factors can serve as protective. Such knowledge is crucial for the development of efforts to improve working conditions at schools for Swedish teachers. On an individual teacher level, of course, it may help those teachers experiencing high levels of stress. Moreover, it may also be valuable for students’ school well-being, since there is empirical evidence on the links between teacher stress and students’ school well-being [6]. While prior research has mainly focused on the association between teachers’ own experiences of their work environment and stress-related outcomes, it is also possible that colleagues’ perception of the work environment and their possibilities for dealing with work-related stress contribute to influencing individual teachers’ stress. Furthermore, although there is a body of international research on working conditions and stress among teachers [1,2,7,8], surprisingly few Swedish studies have addressed the topic, which can be considered problematic, given the high and increased stress levels reported by Swedish teachers. The educational reforms that have been implemented in Sweden over the past 25 years seem to have affected the stress level of Swedish teachers negatively. Few other countries’ educational systems have undergone such dramatic changes. The educational reforms implemented in the 1990s were, in many respects, poorly implemented [3], causing prolonged frustration among teachers, along with a substantially increased administrative burden [3–5]. The administrative burden together with increased workload and time pressure are also emphasised as the most important causes of perceived stress among teachers in all OECD countries [2]. The past years’ statistics show that teachers are among the most stressed occupational groups in Sweden today, with a higher workload, less feedback and support from superiors, less perceived control over their work situation and higher sick-leave rates (especially from depression and burnout syndrome) compared to other non-manual occupational groups [3–5,9–11]. The situation of high and increased levels of reported teacher stress in Sweden together with few national studies addressing this topic makes it especially relevant to target this topic in Sweden. Using survey data collected among 2732 teachers in 205 senior-level and upper secondary schools in Stockholm, Sweden, in 2014 and 2016, the present study sought to analyse individual and contextual expressions of psychosocial working conditions among teachers and their associations with stress-related outcomes.

### The job strain model

Research on the psychosocial work environment is extensive, and different theoretical models have been used to explain associations between working conditions and stress and stress-related outcomes. One commonly studied theoretical model is the Demand-Control Model [12], suggesting that high demands and low control in the workplace give rise to high job strain, which in turn is associated with stress-related ill-health. It is well documented that being exposed to high psychological demands and low job control repeatedly or over a long period of time is associated with poor health outcomes [13,14]. A further developed model is the Job Demand-Control-Support (JDCS) model [15,16], in which social support is added as a third dimension to demand and control. With regards to the role of social support in the JDCS model, two hypotheses have been derived. The iso-strain hypothesis suggests that high demands and low control combined with low social support (or isolation) adversely affect health. The buffer hypothesis states that social support can also be understood as a buffering factor that may moderate the negative impact of high strain [17]. Another model is the Job Demands-Resources (JDR) model, suggesting that job resources can buffer the impact of job demands on strain. Just as there are different theoretical models, there are a variety of tools and questionnaires to assess working conditions. One example is the Copenhagen Psychosocial Questionnaire (COPSOQ) which is used for mapping the psychosocial work environment. Another questionnaire that is often used for this purpose, but targeting fewer dimensions, is the Demand-Control Questionnaire (DCQ) [18,19], which served as the base for this study.

### Sense of coherence

Traditionally, identifying risk factors has dominated the research field of work-related health, but more recently a salutogenic approach has become more prevalent [20]. The salutogenic perspective (introduced by Antonovsky) suggests that it is important to look at individuals’ own capacity and their resources to create health. The sense of coherence (SOC) concept constitutes the essence of this salutogenic perspective and refers mainly to individuals’ tendency to understand their life situation (comprehensibility), how they think they can handle their life situation (manageability) and whether they find their life situation meaningful [21,22]. The basic idea of SOC means that individuals who report high values on these dimensions are better equipped for daily stressors and thus do not experience their environment as stressful to the same extent as individuals with low SOC values, as reported in previous studies [23–26]. High levels of SOC have in previous studies been shown to be a positive predictor of mental health among teachers [27,28]. Even if SOC is defined on the individual level as a tendency to perceive life as comprehensible and meaningful and also to consider oneself capable of managing problems – and therefore to be better able to deal successfully with health-threatening stressful situations of everyday life – SOC may also be seen as a collective resource, as argued by Antonovsky and as operationalised in previous studies [25]. The relevance of SOC as a group characteristic may be understood in terms of an effective resource when it comes to influencing collective stress factors in the workplace [25]. Even though SOC is not a work-related construct, previous studies have investigated individual-level SOC in relation to the job strain model, showing that SOC is of importance in the association between job strain and stress-related ill-health [29,30]. In this study, SOC will be used as an individual item as well as a proxy of school contextual SOC in order to explore its role in the association with work-related stress among teachers.

### Contextual factors

Previous research shows that work-related stress is not only caused by individual experiences, but that it can also spill over to others within a workplace, meaning that collective experiences within a workplace also may affect the individuals within that setting [31,32]. Prior studies have also demonstrated how work-related factors at the workplace level interact with the individual, indicating that contextual features of the work environment cause stress to the individual [33–35]. Accordingly, it seems important to examine such contextual factors by taking into account colleagues’ experienced working conditions and the collective SOC and how these factors are associated with the individual teacher’s experience of stress and stress-related complaints, regardless of how he or she estimates the working conditions him/herself.

### Aim of the study

The aim of the study was to investigate how teachers’ reports of high job strain (i.e. high demands and low control) and SOC, as well as the proportions of colleagues who reported high strain and high SOC, were associated with perceived stress and depressed mood at the individual level.

## Methods

The data were derived from the Stockholm Teacher Survey of 2014 and 2016. These cross-sectional surveys were carried out by our research group through a web-based questionnaire targeting teachers in the Stockholm municipality. The questionnaire included questions related to SOC and a wide range of questions on work-related issues such as psychological demands, job control, stress and stress-related complaints. The questions used to assess working conditions derive from the DCQ [18,19] and have previously been used, for example, in the longitudinal SLOSH study [36], while the questions related to stress and depressed mood have previously been used in research related to teacher stress [6]. In 2014, the sample frame included 2374 senior-level teachers (grades 7–9), of whom 1287 participated (response rate 54.2%). In 2016, the sample frame included 2324 senior-level teachers, of whom 1247 participated (response rate 53.7%), and 2443 upper secondary school teachers, of whom 1414 participated (response rate 57.9%). Thus, the total response rate was 55.2% (3948/7147). Schools with six or fewer participating teachers were excluded from the study sample (*n*=308), just as teachers with missing information on any of the variables used in the analysis (*n*=908), meaning that the final study sample comprised 2732 teachers distributed across 205 school units.

### Dependent variables

Teacher stress was measured by an index comprised of three items derived from asking teachers to what extent they: ‘have days when they constantly feel tense or wound up’, ‘have days when they feel pressured, nearly more than they can manage’ and ‘have days when they constantly feel stressed’. The response categories were ‘not at all’, ‘sometimes’, ‘fairly often’ and ‘nearly always’ which were given numeric values 1–4, resulting in a sum index ranging between 3 and 12, with higher values indicating more stress (Cronbach’s α=0.88).

Teacher depressed mood was captured by the question ‘To what extent in the past week have you experienced. . .?’ followed by six items: ‘fatigue or lack of energy’, ‘depressed mood’, ‘blaming yourself for things’, ‘excessive worry’, ‘lack of interest’ and ‘that everything feels exhausting’. The response categories were ‘not at all’, ‘a little’, ‘moderately’, ‘fairly’ and ‘very much’. These were given numeric values 1–5, resulting in a sum index ranging between 6 and 30 (Cronbach’s α=0.91). The Symptom Checklist – Core Depression (SCL-CD6) has been widely used in previous research and has been found to be a valid scale for measuring depressed mood [37].

Both indices were standardised (*M*=0, *SD*=1), and the correlation between the two measures was *r*=0.62.

### Independent variables

#### Individual level

Psychological demands was constructed from five questions derived from the DCQ: ‘Does your job require you to work fast?’, ‘Does your job require you to work very hard?’, ‘Does your job require too much effort?’, ‘Do you often experience conflicting demands in your work?’ and ‘Do you have adequate time to complete your job tasks?’. The response categories were ‘often’, ‘sometimes’, ‘rarely’ and ‘never’ and were given numeric values ranging from 4 to 1 (the last item reversely coded), resulting in a sum index ranging between 5 and 20, with higher values indicating higher demands (Cronbach’s α=0.79).

Job control was measured by six questions, also derived from the DCQ: ‘Do you get to learn new things in your job?’, ‘Does your work involve doing the same thing over and over again?’, ‘Do you have the freedom to decide how your work tasks will be performed?’, ‘Do you have the freedom to decide which work tasks you will do?’, ‘Does your work require skilfulness?’ and ‘Does your work demand ingenuity?’. The response categories were ‘often’, ‘sometimes’, ‘rarely’ and ‘never’ and were given numeric values ranging from 4 to 1 (the second item reversely coded), resulting in a sum index ranging between 6 and 24 (Cronbach’s α=0.52).

Job strain is a combination variable of the individual’s reports of psychological demands and job control, as described above. To define the variable, the median values for the demand and the control scale, respectively, have been used. The median value for demands is 16 (scale ranging from 5 to 20), and for control it is 20 (scale ranging from 6 to 24). As pointed out in previous research [38], no numeric cut-off values have been defined for job strain. Instead, as also originally recognised by Karasek [12], using the median cut points on the two scales is the most common approach. The combination of the two scales results in the classification into four job types: low strain, high strain, active and passive. In this study, high job strain is contrasted to the other groups combined.

SOC was captured by a validated three-item instrument [39]. It was based on the statement ‘Do you usually. . .?’ and the questions ‘. . .feel that things that happen in your daily life are difficult to understand?’ (comprehensibility), ‘. . .find a solution to problems and difficulties that others find hopeless?’ (manageability) and ‘. . .feel that your daily life is a source of personal satisfaction?’ (meaningfulness). The response categories were ‘yes, often’, ‘yes, sometimes’ and ‘no’. The item measuring comprehensibility was coded from 1 to 3, and the items measuring meaningfulness and manageability were coded from 3 to 1, resulting in a sum index ranging between 3 and 9 (Cronbach’s α=0.51). In accordance with earlier studies [28,39], high SOC was defined by a cut-off of ⩾7.

#### School level

Proportion of teachers with high job strain was defined as the percentage of teachers in each school who reported high strain. Proportion of teachers with high SOC was defined as the percentage of teachers in each school who reported high SOC.

#### Control variables

A set of control variables were included: sex (female or male), years in profession, study year (2014 or 2016), school level (senior or upper secondary level) and school type (public or independent).

### Ethics

The Stockholm Teacher Survey has been approved by the Regional Ethical Review Board of Stockholm (2013/2188-31/5; 2015/1827-31/5).

### Statistical method

The method used was multilevel analysis which handles hierarchical data [40], for example teachers who are nested in schools. Two-level random intercept linear regression models were performed using Stata’s *xtmixed* command. In an initial step, we performed two-level (because of the data structure) linear regressions to assess the associations between teachers’ reports of psychological demands, job control and SOC, on the one hand, and their perceived stress and depressed mood, on the other. Subsequently, we performed two-level linear regression analyses examining low/high strain combined with low/high SOC, as well as the contextual school-level variables: proportion of teachers reporting high strain and the proportion of teachers reporting high SOC, and their associations with stress and depressed mood. These analyses were performed in a series of models. First, an empty model was estimated in order to investigate the variation between schools in each of the dependent variables. This allows the variation in the dependent variables to be separated into two components: teachers and schools. Model 1 shows the estimates for different combinations of low/high strain and low/high SOC, where low strain and high SOC serve as the reference category. In model 2, the contextual school-level variable proportion of teachers with high strain was added, while model 3 instead added the proportion of teachers with high SOC. Finally, in model 4, all variables were mutually adjusted for. All models were adjusted for sex, age, years in profession, study year, school level and school type. For each model, the intraclass correlation (ICC) is reported, which provides information about how much of the total variance in the dependent variable that is accounted for by the school level rather than the individual teacher level [41]. In order to assess the model improvement when adding school-level variables, the likelihood ratio test was performed, where model 1 was compared to the others. *R*^2^ for the individual and school level was calculated by using the *mltrsq* command in Stata. Bryk/Raudenbush estimates presented in Table III indicate the explained proportion of variance at each level and are based on the reduction of unexplained variance when predictors are added to the model. Furthermore, we checked the proportion of shared variance in our independent variables of interest. The ICC was 9.3% for demands, 6.2% for control and 1.9% for SOC.

## Results

Table I presents descriptive statistics of the study sample.

**Table I.** Descriptive statistics of the study sample (*N*=2732 teachers distributed over 205 schools).

*Individual level*				
Perceived stress^a^	7.05	2.23	3	12
Depressed mood^a^	13.79	5.81	6	30
Psychological demands^a^	15.66	2.63	5	20
Job control^a^	20.23	1.92	9	24
Sense of coherence^a^	7.38	1.20	3	9
Age^a^	43.61	11.35	18	76
Low strain, high SOC^b^	1526	55.9		
Low strain, low SOC^b^	523	19.1		
High strain, high SOC^b^	554	20.3		
High strain, low SOC^b^	129	4.7		
Sex				
Female^b^	1697	62.1		
Male^b^	1035	37.9		
Study year				
2014^b^	946	34.6		
2016^b^	1786	65.4		
Years in profession				
<1 year^b^	11	0.4		
15 years^b^	392	14.4		
6–10 years^b^	549	20.1		
11–15 years^b^	604	22.1		
16–20 years^b^	462	16.9		
⩾21 years^b^	714	26.1		
*School level*				
% teachers with high strain^a^	25.0	2.7	18	26
% teachers with high SOC^a^	76.5	11.6	37	100
School level				
Senior^b^	1741	63.7		
Upper secondary^b^	991	36.3		
School type				
Public^b^	1880	68.8		
Independent^b^	852	31.2		

a Data shown as *M, SD*, min and max.

b Data shown as *n* and %.

Estimates from two-level linear regressions presenting the associations between psychological demands, job control and SOC with stress and depressed mood are presented in Table II. Here, the estimates for psychological demands, job control and SOC are presented to demonstrate their independent associations with stress and depressed mood, while in Table III, estimates for low and high strain as well as low and high SOC are presented. In both the unadjusted and the adjusted analyses in Table II, psychological demands showed positive and statistically significant associations with both outcomes, while job control and SOC showed negative and statistically significant associations. When all three predictors were considered simultaneously in the adjusted model, all estimates remained significant, but it can be noted that especially the estimates for job control were attenuated.

**Table II.** Results from two-level linear regression models, adjusted for sex, age, years in profession, study year, school level and school type (*N*=2732; unstandardized values).

	Unadjusted^a^		Adjusted^b^	
	Coefficient	95% CI	Coefficient	95% CI
*Perceived stress*				
Psychological demands	0.53***	0.50, 0.55	0.48***	0.45, 0.50
Job control	−0.28***	−0.32, −0.23	−0.08***	−0.11, −0.05
Sense of coherence	−0.62***	−0.68, −0.55	−0.37***	−0.42, −0.31
*Depressed mood*				
Psychological demands	0.98***	0.90, 1.06	0.77***	0.70, 0.84
Job control	−0.72***	−0.83, −0.61	−0.16**	−0.25, −0.06
Sense of coherence	−2.31***	−2.47, −2.16	−1.89***	−2.04, −1.73

*** *p*<0.001; **p<0.01; *p<0.05.

a Psychological demands, job control and sense of coherence included one at a time.

b Psychological demands, job control and sense of coherence mutually adjusted.

CI: confidence interval.

**Table III.** Results from two-level linear regression models, adjusted for sex, age, years in profession, study year, school level and school type (*N*=2732 teachers distributed over 205 schools; unstandardized values).

	Empty model		Model 1		Model 2		Model 3		Model 4	
			Coefficient	95% CI	Coefficient	95% CI	Coefficient	95% CI	Coefficient	95% CI
*Perceived stress*										
Low strain, high SOC (ref.)			0.00	−	0.00	−	0.00	−	0.00	−
Low strain, low SOC			1.44***	1.24, 1.65	1.44***	1.23, 1.65	1.35***	1.14, 1.57	1.35***	1.14, 1.57
High strain, high SOC			1.00***	0.80, 1.21	1.00***	0.80, 1.21	1.00***	0.79, 1.20	1.00***	0.79, 1.20
High strain, low SOC			2.08***	1.70, 2.45	2.07***	1.70, 2.45	2.00***	1.62, 2.37	1.99***	1.62, 2.37
% teachers with high strain					1.74	−2.07, 5.55			1.55	−2.14, 5.25
% teachers with high SOC							−1.54***	−2.33, −0.75	−1.53***	−2.31, −0.74
School-level variance (*SE*)	0.24***	(0.06)	0.13	(0.05)	0.13	(0.05)	0.10	(0.04)	0.10	(0.04)
ICC	4.8%		3.0%		2.9%		2.4%		2.3%	
*R*^2^ individual/*R*^2^ school			0.11/0.46		0.11/0.47		0.12/0.57		0.12/0.58	
Model improvement					*p*=0.372		*p*<0.001		*p*<0.001	
*Depressed mood*										
Low strain, high SOC (ref.)			0.00	−	0.00	−	0.00	−	0.00	−
Low strain, low SOC			5.17***	4.65, 5.70	5.17***	4.65, 5.70	4.98***	4.44, 5.52	4.98***	4.44, 5.52
High strain, high SOC			1.18***	0.66, 1.70	1.18***	0.66, 1.70	1.17***	0.65, 1.69	1.16***	0.64, 1.68
High strain, low SOC			6.81***	5.85, 7.76	6.79***	5.84, 7.74	6.62***	5.66, 7.58	6.61***	5.65, 7.57
% teachers with high strain					5.91	−2.69, 14.52			5.58	−2.91, 14.08
% teachers with high SOC							−2.71**	−4.56, −0.86	−2.67**	−4.51, −0.83
School-level variance (*SE*)	0.69***	(0.32)	0.21	(0.20)	0.18	(0.20)	0.16	(0.20)	0.13	(0.19)
ICC	2.1%		0.8%		0.7%		0.6%		0.5%	
*R*^2^ individual/*R*^2^ school			0.16/0.69		0.16/0.74		0.16/0.77		0.16/0.81	
Model improvement					*p*=0.181		*p*=0.004		*p*=0.008	

*** *p*<0.001; **p<0.01; *p<0.05.

SOC: sense of coherence; SE: standard error; ICC: intraclass correlation.

The results of the two-level linear regression analyses, including combinations of job strain and SOC as well as the school-level contextual variables, are presented in Table III. The empty models demonstrate a statistically significant variation across schools in perceived stress and depressed mood among teachers. Next, in order to investigate the role of SOC in the associations between job strain and our stress-related outcomes, analyses of a variable combining high job strain (i.e. high demands and low control) with low versus high SOC were performed (model 1). For both outcomes, high job strain accompanied by low SOC was more strongly associated with the stress-related outcomes than high job strain accompanied by high SOC. Those differences were statistically significant across both outcomes and models, not presented in Table III. Likewise, low job strain accompanied by low SOC was more strongly associated with both outcomes than low job strain accompanied by high SOC. Those differences were also statistically significant across both outcomes.

Next, to assess whether colleagues reporting high job strain and high SOC were associated with stress and depressed mood at the individual level, we included these contextual school-level measures, with results reported in models 2–4. The proportion of colleagues reporting high job strain was not significantly associated with higher perceived individual stress or depressed mood (model 2), and the model improvement was not significant when adding this school-level variable.

The proportion of teachers in the same school reporting high SOC was linked with lower levels of stress (*b*=−1.54, *p*<0.001) and depressed mood (*b*=−2.71, *p*=0.004; model 3), and model fit was significantly improved for model 3 (*p*<0.001 for stress and *p*=0.004 for depressed mood) when this contextual variable was included. When mutually adjusting for both contextual school-level measures (model 4), the associations between high SOC among colleagues and stress and depressed mood remained robust and statistically significant. Furthermore, it can be noted that there are relatively small differences in the explained variance (*R*^2^) between the different models. In the case of perceived stress, the *R*^2^ at the school level increased from 0.46 to 0.57, and in the case of depressed mood, it increased from 0.69 to 0.77 when introducing school-level SOC. The relatively high *R*^2^ at the school level may seem considerable, but the overall variation derived from the school level in the empty model is only 4.8% for perceived stress and 2.1% for depressed mood, which is why it is important to emphasise that the greatest variation in perceived stress and depressed mood is nevertheless due to differences at the individual level.

## Discussion

This study examined the associations between teachers’ work environment in the form of experienced job strain and SOC with stress-related outcomes in terms of perceived stress and depressed mood. Further, the study investigated whether school contextual conditions (i.e. the proportion of colleagues at the school who reported high job strain and high SOC, respectively) were associated with individual teachers’ perceived stress and depressed mood.

The results showed that psychological demands at work were positively associated with stress and depressed mood, and that job control and SOC demonstrated negative associations with both studied outcomes. These results are in line with international research based on the job strain model [2,8,13,14], thus reinforcing previous findings in the field. Likewise, the results of this study coincide with previous research on the associations between SOC and perceived stress [23–28]. Furthermore, the results presented in Table III reveal that high job strain was less strongly linked with perceived stress and depressed mood among individuals with high SOC, indicating that they were better equipped for the daily stressors they are exposed to in their work environment.

With regard to the contextual level, it was demonstrated that the proportion of colleagues reporting high SOC was associated with lower levels of perceived stress and depressed mood, suggesting that this contextual factor may impact on the individual’s perceived stress and function as a contextual protective factor for stress. There are several possible reasons for this. As argued in previous research [25], even though SOC is defined as individual abilities, it may also be seen as a collective resource, which can help to understand the finding of a school-level effect of SOC. First, it can be assumed that if the average SOC in the workplace is high, with colleagues showing a high degree of comprehensibility, manageability of difficult situations and an experience of meaningful work, these group characteristics may spill over to the individual, which is important, as the individual degree of SOC is also a key protective factor. Second, the collective level of SOC can also serve as an effective resource when it comes to counteracting common stressors at the school, which are specific for every workplace. However, it should be emphasised that the amount of variation in perceived stress (ICC=4.8%) and depressed mood (ICC=2.1%) attributed to the school level was rather limited, indicating that individual-level factors are more important. This finding is in line with studies concerning well-being outcomes [42], as well as with data reported by the OECD, showing that on average across the OECD, only 6% of the variance in teachers’ well-being and stress is accounted for by between-school differences [2]. There was less support of an ‘effect’ of the proportion of colleagues reporting high job strain, meaning that we found no support in the data for the assumption that strain can spill over to colleagues within the same workplace.

Concerning the investigated contextual effects, we can, based on the results from this study, partly confirm previous findings that have shown that collective experiences within a workplace may affect the individuals within that workplace [31–33,43]. The wide range of school-level SOC indicates substantial differences in the levels of this contextual aspect across schools. This implied a lack of precision in the results due to wide confidence intervals, and hence further studies are needed to corroborate the findings.

In line with the job strain model [12] and the results from this study, one can think of several different initiatives in order to reduce the levels of stress among teachers, for example by reducing psychological demands in teachers’ daily work and by increasing their level of job control. This could be done by reducing the administrative burden, the demands for documentation and the degree of external control, which are factors that have previously been reported to contribute to increased stress among teachers [2], as well as by strengthening the autonomy of the teaching profession. Furthermore, our results indicate that the collective SOC in the workplace is relevant, even though the individual level of SOC is also of importance. Developing the working environment towards being more comprehensible and manageable and thereby contributing to strengthen experiences of meaningfulness may lead to a better experience of SOC in the workplace.

One major strength of the study is that we analysed both individual and contextual aspects of teachers’ work environment using well-conceptualised measures of both independent and dependent variables. Another strength is that we used two separate measures for teachers’ stress and depressed mood. Even though they were strongly correlated, together they provide a clearer picture than what just one of them would do. However, it should be acknowledged that the internal consistency for the independent variables control and SOC was relatively low.

The response rate of 55% can be regarded as a limitation. It is possible that non-participating teachers experienced higher levels of stress than those within the study sample, but it is also possible that there is a selection bias among the non-participants towards the less stressed. However, we do not know if any such bias exists or in what possible way this would have affected our findings. Furthermore, among those who participated, there was also item non-response. To investigate possible bias between the teachers in the study sample and the total number of responding teachers, mean values of all variables of interest were compared between these groups, and no noteworthy differences were found.

Another limitation is the fact that the data are cross-sectional in nature, meaning that we cannot make any claims about causality with support in our data. Longitudinal data are desirable for future research. Furthermore, data derive from two waves of cross-sectional data collection, and because the data are anonymised, we have no opportunity to check whether the same teacher participated in both collections, which is considered a limitation. However, stratified analyses were performed separately for senior-level teachers from 2014 and 2016, as well as upper secondary level teachers separately. The associations examined pointed at the same direction for all these groups but did not always reach statistical significance, which may be due to lower statistical power. Finally, while the study was performed among teachers in Stockholm, generalisability to other geographical areas and educational systems should be made with caution. To corroborate the findings, studies of individual and contextual expressions of teacher job strain and SOC in relation to teachers’ stress-related health outcomes in other geographical and educational contexts are recommended. Future studies could use other theoretical models, such as the Effort-Reward Imbalance model or the JDR model, to explore the relationships between the work environment, SOC and stress. Likewise, other questionnaires, such as the COPSOQ or the Job Content Questionnaire, could be used to assess working conditions in order to study individual and contextual associations between working conditions and stress-related health among teachers.

## Conclusions

This study contributes to increased knowledge about the links between psychosocial work-related factors, SOC and stress-related outcomes among teachers. The results showed that high job strain was related to higher levels of stress and depressed mood among teachers in Stockholm municipality, but that high SOC seems to be a protective factor. Furthermore, the proportion of colleagues reporting high SOC was related to less individual stress and depressed mood, suggesting a protective effect of school-level collective SOC.
